# The contributory role of an autistic presentation to miscarriage of justice in a high-profile murder case in New Zealand

**DOI:** 10.1080/13218719.2023.2260846

**Published:** 2024-01-10

**Authors:** C. S. Allely, T. McKinnel, N. Chisnall

**Affiliations:** a School of Health and Society, University of Salford, Greater Manchester, UK; b Gillberg Neuropsychiatry Centre, Sahlgrenska Academy, University of Gothenburg, Gothenburg, Sweden; c Investigator and Director, Zavest, Auckland, New Zealand; d Barrister, Auckland, New Zealand

**Keywords:** autism spectrum disorder, investigative interviewing, miscarriage of justice

## Abstract

In New Zealand in 1985, Mr Alan Hall was convicted of murdering Arthur Easton and spent more than 19 years in prison. He was finally acquitted by the Supreme Court in 2022. In 2019, Mr Hall was diagnosed with autism spectrum disorder (ASD). There are a number of aspects of Mr Hall’s investigative interviews conducted prior to his conviction in 1985 – the questions posed to him and his responses to these – which could be interpreted as being evidence of evasiveness, remorse, lack of empathy and guilt by both the investigating interviewer and the jury and judge when the evidence from this interview was later presented in court. This article discusses how the police approach to interviewing and Alan Hall’s ASD were the catalysts for a tainted investigation, prosecution and conviction. The Crown now accepts that a substantial miscarriage of justice occurred in Mr Hall’s case.

Mr Arthur Easton and his two teenage sons were confronted by an armed intruder in their Papakura home in New Zealand on the evening of 13 October 1985. In the course of the frenzied scuffle that ensued, the intruder stabbed Mr Easton and one of his sons before fleeing on foot, leaving behind his weapon, a bayonet, and the woollen hat he had used to obscure his face. Mr Easton died at the scene, one of the stab wounds having pierced his liver. Mr Hall, then aged 23, came to police attention two months after Mr Easton’s murder due to his apparent ownership of both a bayonet and a woollen hat. Mr Hall was subjected to lengthy interviews by police on 11 December 1985 and 16 December 1985. During the interviews he told police that he thought both items left at the murder scene were his. The bayonet was a Swedish Army item. A total of 300 were imported into New Zealand two to three years before the homicide. Mr Hall had purchased one of the bayonets imported on 27 June 1983. The hat purchased by Mr Hall’s brother in about 1982 was one of only 50 manufactured and sold in New Zealand. He said he had not seen it since 1983 and said that both his brothers, Alan and Geoff, had borrowed it from time to time. He consistently denied ever entering the Easton property. However, over time he gave conflicting accounts of when he had last seen these items and what had happened to them. He further accepted that he had been out walking near the Easton house on the night of the murder.

In April 1986 Mr Hall was charged with murdering Arthur Easton and intentionally wounding Brendon Easton. After a trial by jury in September 1986, Mr Hall was convicted of the murder of Arthur Easton and of wounding Brendon Easton. He was sentenced to life imprisonment.

Maintaining his innocence, Mr Hall sought leave to appeal against conviction to the Court of Appeal. His appeal was dismissed in 1987. Mr Hall applied unsuccessfully on three occasions, between 1988 and 1991, for the exercise of the royal prerogative of mercy. Mr Hall was released on parole in November 1994 and lived in the community for the following 18 years before being recalled to prison on an interim basis in May 2012 for breaching a condition of his parole. Recall became final in June 2012. Mr Hall was again released on parole in February 2022, four months before his appeal was to be heard in the Supreme Court.

On 8 June 2022, 36 years after Alan Hall was convicted of murder and wounding, the Supreme Court heard his second appeal (Hall v R, 2022). The grounds of appeal focused on a number of issues – most notably, the suggestion that Mr Hall and the jury had been deceived by the prosecution, that the prosecution had failed to disclose material evidence, and Mr Hall’s dealings with police, in particular the manner in which the police had interviewed him.

## The importance of Mr Hall’s interviews with police

Following Mr Hall’s interviews with police on 11 and 16 December 1985, he became the prime suspect for Mr Easton’s murder. In his interviews, Mr Hall told police that the bayonet and the woollen hat left at the murder scene were likely his, and that he had been in the area on the night of the attack. However, he adamantly denied any involvement in the attack on Mr Easton, or knowing anything about it. In the course of his lengthy interviews with police, Mr Hall gave conflicting accounts of the whereabouts of the bayonet and woollen hat. In summing up the case to the jury, the presiding Judge referred to the ‘truth or falsity’ of those explanations as ‘an important, if not a crucial issue in this case’. It is important to note that the Crown’s case hinged on the fact that Mr Hall appeared to lack credibility, and his behaviour was consistent with guilt because he provided various explanations. Mr Hall’s statements to police were appropriately characterised by Mr Hall’s counsel in the Supreme Court as of ‘critical importance’ to the Crown case.

There are several parts of Mr Hall’s interview: the questions posed to him and his responses to these, which could be interpreted as being evidence of evasiveness, remorse, lack of empathy and guilt by both the investigating interviewer, and later the jury, judge and appellate court when the evidence from this interview was later presented or relied upon in court.

Following Mr Hall’s interviews, police were confronted with a substantial incongruence arising from eyewitness descriptions of the offender, relative to Mr Hall’s appearance. Mr Easton’s killer had been described by eyewitnesses as a six-foot-tall male with dark skin, likely right-handed and of sturdy build. Mr Hall was five foot seven inches tall, light skinned, left-handed and of slight build. Despite the difficulties with the identification evidence, Mr Hall was arrested and charged with murder and wounding in 1985, and his police interviews became the foundation of the prosecution case against him. Mr Hall’s lawyer argued at trial in 1986 and at his appeal in 1987 that Mr Hall was ‘intellectually backward’, and that his backwardness explained the apparently concerning features of his police interviews. What was not then known, nor until 2019, was that Mr Hall had autism spectrum disorder.

How a diagnosis of autism spectrum disorder may impact on the response and manner of the individual during an investigation is discussed further in this paper, followed by a brief analysis of how Mr Hall’s interviews altered the course of the police investigation, inducing acts of police and prosecutor misconduct, ultimately leading to an egregious miscarriage of justice.

## Diagnosis of autism spectrum disorder

In 2019, as Mr Hall’s legal team prepared a final appeal to the Supreme Court, a clinical psychologist was engaged to assess Mr Hall. At the conclusion of the assessment, he was diagnosed with autism spectrum disorder (ASD), a neurodevelopmental disorder characterised by reciprocal social interaction and communication impairments and also restricted repetitive behaviours. The fifth edition of *The Diagnostic and Statistical Manual* (DSM–V; American Psychiatric Association, APA, 2013) characterises two core areas of impairment in ASD. These are: (a) ‘persistent deficits in social communication and social interaction’ and (b) ‘restricted, repetitive patterns of behavior, interests, or activities’ (APA, 2013). Repetitive behaviours and restricted interests (RBRIs) characterise behaviours such as repetitive motor movements, sensory reactions, rituals, routines and restricted interests.

Regarding vulnerability during police investigative interviewing, some individuals with ASD may not necessarily be recognised as immediately vulnerable due to their apparent competent use of language and by virtue of the fact that they appear to be intellectually capable. However, individuals with an ASD may experience considerable difficulties in being able to understand and cope with police demands despite these apparent abilities. High levels of distress in the context of the closed social situation of an investigative interview is also common in individuals with ASD (North et al., 2008).

## Memory

Research shows that memory impairment may exist in individuals with ASD, which may make them more vulnerable during an investigative interview (e.g. Bigham et al., 2010; Boucher et al., 2012; Bowler et al., 1997; Maister et al., 2013). Specifically, individuals with ASD frequently have difficulty in recollecting or remembering past personally experienced events and tend to remember fewer of them as well as taking more time to do this than those without ASD (Crane et al., 2012; Goddard et al., 2007). Individuals with ASD tend to have a better memory for semantic and general information. However, they may need more prompting in order to retrieve specific episodes (Crane & Maras, 2018). There is also a tendency for individuals with ASD to rely on feelings of familiarity in order to guide their memory much more than individuals without ASD (e.g. Bowler et al., 2000; see also Johnson et al., 2018; Maras & Bowler, 2012). Questions that can be particularly challenging for individuals with ASD include those that ask ‘when’ an event occurred, or differentiating details between episodes, because autism impacts on memory retrieval (The Advocate’s Gateway, 2016).

During his interrogation, Mr Hall demonstrated memory for what he was wearing on the night in question but has difficulty in recalling other aspects of the evening, such as the timing of events. This would be considered by the police, and ultimately the court, as evidence of guilt or that he had something to hide. However, with individuals with ASD, like Mr Hall, these seemingly inconsistencies are common. For instance, individuals with ASD have the tendency to recall fewer social details of an event (e.g. relating to people and actions), although their recall of details that are non-social in nature (such as objects and surroundings) is usually good (The Advocate’s Gateway, 2015). Additionally, individuals with ASD are often impaired or have difficulty in their ability to recall events in a sequential manner (a clearly sequenced narrative of events) and with sufficient detail. This difficulty can make them appear to be uncooperative and even non-responsive when they are being questioned by police, lawyers or judges (see also Kroncke et al., 2016). Had Mr Hall’s diagnosis of ASD been known at the time of his police interrogation (and the knowledge we now have regarding difficulty in recalling events in a sequential manner in individuals with ASD), it would have provided some explanation as to his difficulty in recalling the events of the night in question, and the necessary and appropriate measures could have been introduced. For instance, recently, guidelines suggest that when interviewing someone with ASD, it is important to use communication aids that help an individual to explain what happened. Some of these communication aids include visual timelines to support the individual with ASD in accurately sequencing events (The Advocate’s Gateway, 2016).

There are a number of instances during his police interrogation where Mr Hall demonstrates confusion, difficulty and some inconsistency in his description of what he was doing on the evening that Arthur Easton was murdered. Given that we now know that Mr Hall has a diagnosis of ASD, in addition to the relatively recent research that now shows that individuals with ASD can have difficulties in memory and the temporal ordering of events that took place, it sheds light on his confusion regarding the timing of events, etc, during the night in question. Had such a diagnosis and related research and information been available to the police (and subsequently made available during the court proceedings), it may have provided an alternative explanation for why Mr Hall was responding in the way that he was – rather than assuming it was evidence of evasiveness and guilt. Below is one example demonstrating Mr Hall’s confusion when questioned:

Q. ‘Allen, that is a 15 minute walk at the most. You were away for an hour at least, maybe an hour and ten minutes. Where were you?’

A. ‘I was walking’.

Q. ‘I told you that is about a 15 minute walk. What were you doing for the other 45 or 55 minutes?’

A. ‘I don’t know what time I left. It might have been 5 to eight, I don’t know. I might have gone to the toilet or something first. That’s right, Benson was still on and I went to the toilet. I didn’t see the end of Benson that’s all’.

Q. ‘How long did you stay in the toilet?’

A. ‘I don’t know’.

Q. ‘You have told the other Detectives that you went walking after Disneyland at first, then they asked you was it raining, so you said you watched a bit of Benson. Now you tell me you could have left at 5 to 8.

Where were you for 45 to 55 minutes Alan?’

Given the growing body of evidence indicating the various memory difficulties in individuals with ASD coupled with Mr Hall’s subsequent formal diagnosis of ASD, it is important that such responses are reconsidered. Presented outwith the context of a diagnosis of ASD and its impacts on memory, such responses would be considered evidence of guilt. Another difficulty with some individuals with ASD is estimating ‘how long’ specific events lasted (The Advocate’s Gateway, 2016). It has been argued that disorders in timing and/or time perception may be a key feature, or cause, of some of the behavioural and cognitive impairments associated with ASD (e.g. Allman, 2011; Allman & DeLeon, 2009; Allman et al., 2011). Issues with timing and/or the perception of time can potentially explain Mr Hall’s response to one of the questions during the police interrogation regarding what he was doing on the night that Arthur Easton was murdered:

Q. ‘How long did you stay in the toilet?’

A. ‘I don’t know’.

## Lack of outward emotional expression and apparent inappropriate expressions/behaviours

It is important to recognise and understand that individuals with an ASD may often speak in a monotone way and lack emotional intonation (this was the case for Mr Hall as these features of ASD were observed by the Consultant Clinical Psychologist Tanya Breen who evaluated Mr Hall and diagnosed him with ASD in 2019 – see Breen, 2019). It is also important to recognise that they can sometimes be quite blunt in their comments, which may be the result of their very literal cognitive style or interpretation of information (Murphy, 2018). Throughout the lengthy police interrogation of Mr Hall, a number of notes regarding Mr Hall’s lack of emotion are made by the interviewing officers. Such behaviour in a suspect or defendant would almost certainly be perceived as evidence that the individual is callous and indifferent to the consequences of their alleged offending behaviour. Such behavioural displays may be viewed by police as confirmation of their hypothesis regarding the person’s guilt (Brewer & Young, 2015). Mr Hall was interviewed by police on the 11 and 16 December 1985 regarding the homicide of Mr Easton. On numerous occasions throughout these interviews with Mr Hall, there are notes and observations made regarding Mr Hall’s lack of emotion. However, what is not considered during these observations is the now well-established findings that many individuals with ASD are impaired in their ability to appreciate the subjective experiences of others (which is usually referred to as an impaired theory of mind, ToM, in the literature – see Jones et al., 2018). As a result of this impairment, the individual with ASD may not display any outward expressions of empathy or intersubjective resonance, which may make them appear to be cold and calculating. This apparent lack of feeling exhibited by the interviewed suspect or defendant with ASD can have a negative impact on them. For instance, it can make people (police, jurors, judge) view them as being guilty and lacking in any remorse. Certainly, Mr Hall’s presentation, consistent with the above, would have been viewed negatively during his investigative interviews with the police and in the court if it was not clearly explained the reason why he may be presenting in this manner (which it was not at this point as the diagnosis of ASD came much later when he was imprisoned). A few examples in the investigative interviews where the police note Mr Hall’s lack of emotion are given below.

Example 1.

Q. But you are involved Allan [police misspelled Mr Hall’s name]. That is your bayonet and hat.

You weren’t at home at the time Mr EASTON was killed. What do you expect us to think?

A. Yes I suppose it does look suspicious.

(Show Alan photo of body of EASTON. Nothing said).

A. Is that him the man that was killed?

Q. Yes. That’s Mr EASTON.

A. I hope you get the bastard that did that.

Q. Yes. Have you seen that man before?

A. No. I saw a photo on the wall when the other Detectives talked to me.

Q. Have you seen this man before?

A. No. (No emotion at all).

Just flick through a couple of photos.

A. Is that all you want me to see.

Q. Mr EASTON didn’t deserve to die Alan. He has got two boys and a daughter. He’s a family man just like your Dad. It was your bayonet that did this to him Alan. How did your bayonet get over to Mr EASTON’S house.’

A. It was stolen. How should I know.

(Flick more photos).

(He started to get nervous and move in his seat).

A. Is that all you want me to see. I have seen dead bodies before.

My father has just died. I saw him.

Q. Do these photos affect you Alan?

A. No.

(No emotion again).

Example 2.

Q. I told you about the man who saw the person running across Clevedon Road. He made up an identikit picture. I have had a look at your passport photo. It looks a lot like you. Did you do this murder Alan?

A. No. I told you I was not brought up to do things like that.

(No emotion at all).

In these two examples, Mr Hall exhibits no outward expression of emotion that you would typically expect to see. Such an outward lack of emotion would be considered to be evidence of guilt and lack of remorse. However, such behaviour considered within the context of a diagnosis of ASD provides an explanation for the behaviour and provides understanding into why the individual may be presenting in the way that they are. It is important to emphasise that while individuals with ASD may exhibit no emotional expression it does not necessarily reflect what they are actually feeling inside (Allely & Cooper, 2017). In her Clinical Psychology Report, Consultant Clinical Psychologist Tanya Breen also made a note regarding Mr Hall’s voice and how it lacked emotional expression (Breen, 2019, pp. 8). Later, in her report, Breen states that Mr Hall ‘ . . . seemed emotionally ‘flat’, and had great difficult talking about thoughts or feelings’ (Breen, 2019, p. 23 – this quote is written as found in her report). For completeness, the Supreme Court acknowledged that, in Example 2, the interviewing officer misled Mr Hall, as the man shown in the identikit bore no resemblance to him.

## Issues with compliance

Research has found that, in some contexts, individuals with ASD may exhibit greater levels of compliance, eagerness to please and avoidance of confrontation (Chandler et al., 2019; North et al., 2008). Individuals with ASD are at increased risk of complying with the pressures of an investigative interview context, which may result in, for example, the individual making statements that are erroneous and self-incriminating (Gudjonsson, 2003) or respond compliantly to the requests and demands of the interviewer (Maras & Bowler, 2012). Individuals with ASD may also be particularly fearful of figures in authority who place them under what they experience as pressure by their authoritarian manner and their style of questioning (see Freckelton, 2011; Freckelton & Selby, 2009). One potential explanation for why some individuals with ASD may be predisposed towards compliance with a desire to please the interviewer is the social skills impairments that frequently result in increased levels of social anxiety (e.g. Kuusikkoet al., 2008; Maras & Bowler, 2012). The issue of compliance is important to consider when Mr Hall was asked ‘You must be a bit tired?’ by police after a number of hours of being interrogated. Mr Hall responded, ‘No, I’m alright’. He may have been tired but have been unwilling to say so in order to please the interviewer and avoid confrontation. An individual with ASD, as a result of higher levels of compliance, may not ask for help or alert the court to the fact that they are struggling to concentrate and need to take a break (The Advocate’s Gateway, 2015). In sum, during an investigative interview, higher levels of compliance may result in the individual with ASD being pressured into agreeing to a statement in order to terminate the interview sooner (Gudjonsson, 2003).

## Presence of paranoia

Mr Hall consistently told the police that he was fearful for his safety and his family’s safety if he was seen to co-operate with the police to identify the perpetrator of the murder of Arthur Easton. This can be seen in the following exchange between Mr Hall and the police officer during the interrogation:

Q. Why didn’t you tell the police that was your bayonet on T.V.?

A. I didn’t want the person who stole it to come back and get me or someone in my family.

Without a thorough understanding of ASD and how it may present, it would be easy to assume that Mr Hall’s explanations of why he did not go to the police here is evidence of his guilt or that he is hiding something. However, when you are interviewing an individual with ASD it is crucial to consider these explanations within the context of ASD. In some contexts, individuals with ASD can display higher levels of trait suspiciousness/tendencies to mistrust others (as measured using the Paranoia Scale, Fenigstein & Vanable, 1992, for instance) than individuals without ASD (e.g. Blackshaw et al., 2001; Maras & Bowler, 2012; North et al., 2008). Individuals with ASD can be more mistrustful of others to a point bordering on paranoia (Freckelton & List, 2009). A variety of contributing factors can explain this, such as: significantly interpersonally isolated, confused or perplexed about social rules, limited in their appreciation of some matters that take place around them, such as expressions of emotion, difficulties in making causal attributions to others’ mental states and their poor understanding of social cues (Blackshaw et al., 2001; Freckelton & List, 2009; Kuusikko et al., 2008; Maras & Bowler, 2012).

## Impaired social communication and interaction

There are a number of times throughout the interview where the interviewing detectives make statements or pose questions to Mr Hall, and he responds in a manner that may be interpreted as being evasive and indicative of guilt. For instance, when the detective asks Mr Hall about the Swedish bayonet:

Interviewer: ‘Tell me about the Swedish bayonet.’

Mr Hall: ‘What about it?’

Mr Hall’s response here may well have been interpreted by the detective as being deliberately difficult or evasive. However, when considered within the context of a diagnosis of ASD there is a completely different explanation for such a response. Consistent with his later diagnosis of ASD, Mr Hall would not have understood exactly what the interviewer was asking of him here. The interviewer needed to be clear and more specific about what exactly he was asking in relation to the Swedish bayonet. For instance, ‘When was the last time you remember seeing your Swedish bayonet?’. The aim of any interview with an individual with an ASD should be to adopt a clear structure and logical order. It is also important to follow a chronological sequence and highlight or signpost any change in subject (Murphy, 2018). There are a number of occasions during the 20 hours of interrogation where there is a marked departure from a clear structure and logical order. For example, there are a few instances when Mr Hall is being questioned about his whereabouts on the night that Arthur Easton was murdered, and then he is suddenly questioned about whether he looked though people’s windows peeping and peering.

It is also strongly recommended that tag questions are avoided, particularly when interviewing individuals with ASD (e.g. The Advocate’s Gateway, 2016). There are a variety of questions that should be avoided with individuals with ASD when they are being questioned; this is just one example. During the 20 hours of interrogation, tag questions are frequently used. For instance,

Q. After the man got murdered, you must have realised something was wrong.

You had a bayonet like the one used.

You knew that didn’t you?

A. Yes.

## Theory of mind impairments

Theory of mind (ToM) refers to the ability to ascribe mental states (beliefs, desires, intentions, emotions, etc.) to oneself and others in order to understand and predict their behaviour. It is well established that poor ToM is one of the key characteristics in individuals with ASD (e.g. Baron-Cohen et al., 1985). This is something that is also important to consider when looking at some of the responses that Mr Hall gives to the police during his interrogation. For instance,

Q. Who took the bayonet and hat Allen?

A. I don’t know.

They just went from my room like I told you.

You don’t believe me either do you.

That other guy doesn’t believe me.

Q. You think about it.

It sounds a bit far fetched don’t you think?

A. I suppose it does.

In the last response made by Mr Hall here (‘I suppose it does’) it gives the impression that he is cold and disinterested in the interview process. However, when looked at in the psychological context of an ASD, the situation is very different. Consistent with ASD, it is possible that Mr Hall is potentially failing to *fully* appreciate the significance of the statement and what the interviewer is implying. Due to an impaired theory of mind (an impaired ability to appreciate the subjective experiences of others) consistent with ASD, he would also fail to appreciate what the interviewer (the police officer) might think of him following a response of this nature. Someone with a non-impaired theory of mind (or someone without a diagnosis of ASD) might think that the other person would see this as being some admission of guilt and would immediately go to great lengths to try and explain it. Mr Hall does not do this.

## Inappropriate expressions

The detective also notes that Mr Hall laughed after one of his responses (considered to be inappropriate behaviour given the context) – see excerpt below.

Interviewer: ‘I would like to know how it got from your place over to Mr EASTON’s house.’

Mr Hall: ‘I don’t know how it got there. I told the other Detectives that it was stolen. They don’t believe me do they? When they come to talk to me one was quiet and the other was heavy. I knew what they were doing. They said I read too many Detective books.’

(Laugh).

Mr Hall laughs here in what is a very tense and serious situation. This seemingly inappropriate behaviour can make the individual being questioned appear cold, remorseless and unempathetic. However, awkward or inappropriate facial expressions or behaviours are often exhibited by individuals with ASD. For example, it is quite common for an individual with ASD to laugh or smile when they are talking about their victim during the court proceedings. However, this outward expression may not reflect what they are feeling internally (Allely & Cooper, 2017). It is something that individuals with ASD may engage in because they do not know what to do in the situation they are in (or understand or know what is expected of them), and it is therefore used as a coping strategy (Allely, 2022).

## Alexithymia

Another important issue that needs to be considered is alexithymia, which refers to an individual’s lack of emotional awareness or, more specifically, an impaired ability or difficulty in identifying and describing feelings and in being able to distinguish feelings from the bodily sensations of emotional arousal. Research has found that alexithymia is highly prevalent in individuals with ASD. Studies have indicated that between 40% and 65% of the ASD population are believed to be alexithymic (e.g. Berthoz & Hill, 2005; Hill et al., 2004). The presence of alexithymia can make an individual appear to others as being cold and lacking in any feeling or emotions in some contexts. This has obvious negative and detrimental consequences in a forensic setting such as in a police interrogation context or when evidence of it is presented during the court proceedings. Mr Hall was not known at the time of the police interrogation, nor during his court proceedings, to have a diagnosis of ASD. Therefore, any evidence he exhibited of having difficulty describing his feelings and emotions would have been misinterpreted. During the interrogation by police, Mr Hall exhibited this difficulty on a few occasions. For instance:

Q. ‘I thought you were close to your Dad?’

A. ‘No I hated him sometimes. He would yell at me all the time.

Sometimes I felt like sticking a knife in his back’.

Q. ‘You don’t mean that, do you Allen?’.

A. ‘No, not really. I was [not] brought up that way. I wasn’t brought up to think like that’.

Q. ‘Why did you say that then?’

A. ‘I don’t know’.

Then later, the police during their interrogation go back to the issue of what Mr Hall felt when his father died:

Q. ‘Were you sad that Dad had died?’

A. ‘No, I was quite happy about it really’.

Q. ‘Why were you happy?’.

A. ‘I don’t know’.

Q. ‘Was it because he was suffering?’

A. ‘No’.

Q. ‘What was wrong with your Dad?’

A. ‘He had diabetes and he had a heart complaint. The Doctor told us that it would only be a matter of time before he went blind and his legs would go. We knew he was going to die’.

Q. ‘Did it upset you when he died?’

A. ‘No’.

Q. ‘Do you miss him?’

A. ‘In some ways yes. I used to do all the heavy lifting for him. He had that glass house out the back. He couldn’t do anything else so he started growing these orchids. That’s all he did’.

Individuals with ASD can be very open, naïve and honest – and not necessarily be aware of the potential negative consequences of this. This is something that Breen observed in Mr Hall and is referred to in her report (see Breen, 2019, pp. 24). In her Clinical Psychology Report, Dr Breen also reports that Mr Hall displayed an ‘inability to talk about emotion and cognition, especially regarding other people’ (Breen, 2019, pp. 24), which is a feature consistent with ASD. She found that when answering questions about thoughts and feelings, Mr Hall would appear confused, and, if the questions were repeated or rephrased, he would look irritated. The difficulty that he had with these types of questions was explored during his second interview with Dr Breen. In this second interview, Mr Hall said that questions about emotions and cognitions was like ‘asking a blind person to see the writing on the wall’. Mr Hall also reported to Dr Breen that he had always experienced great difficulty answering these types of questions. Dr Breen reported that he would tend to say stock phrases like ‘I don’t know’ or ‘I couldn’t say’ or, ‘I don’t know how to answer that’ (Breen, 2019, p. 9). Similarly, when Dr Breen asked Mr Hall how his mother felt when he was imprisoned for murder, Mr Hall said, ‘I wasn’t there, so I couldn’t say’ (Breen, 2019, p. 9). In the psychological report by Kathryn Gilchrist (25 September 2018), she also noted how Mr Hall frequently answered questions with ‘don’t know’. She stated that he appeared to have difficulty answering questions that focused on emotions. This difficulty can be coupled with word-finding difficulties or ‘finding the right words’ to explain what happened that some individuals with ASD have. This might be indicated by pauses, saying ‘I don’t know’ or ‘I can’t explain it’ (The Advocate’s Gateway, 2016). It is worth pointing out here that Mr Hall made such responses very frequently throughout his lengthy interrogation.

## Length of interrogation

Relating also to the issue of compliance detailed earlier, Mr Hall does clearly state that he wishes to stop the interrogation: no breaks were provided despite Mr Hall’s clear requests.

## Misinterpretation or lack of understanding of repetitive interests or behaviours

The jury may also misinterpret or not understand the repetitive interest and/or the particular obsessions that the defendant with ASD might exhibit in court or that might be described during the court proceedings. Many individuals with ASD may shift the focus of the court discussions to something that they want to talk about – which may be one of their interests. Such behaviour may be perceived by the jury as evasiveness – like they have something to hide. Mr Hall exhibited a few repetitive interests, and it important to recognise that this is a feature of his ASD. During the court proceedings, Mr Hall’s possession of bayonets and his interest in military paraphernalia were raised in a potential attempt to malign him because of these particular interests. It is highly likely that Mr Hall’s unusual interest in military paraphernalia was directly related to his ASD symptomology. Such unusual interests are not uncommon in individuals with ASD. Other articles have also noted unusual interests in individuals with ASD when compared to individuals with no diagnosis of ASD. For instance, Hare et al. (1999) found in their study that the group diagnosed as having ASD had significantly more circumscribed interests and repetitive routines than the control groups. Importantly, they found that themes of these circumscribed interests and repetitive routines commonly involved violence, weapons and Nazism (Hare et al., 1999). Unless Mr Hall’s unusual interest was explained to the jury and judge within the context of his ASD, it has the strong potential to be perceived very negatively.

## Issues with time to respond

Many individuals with ASD can have significant mental processing speed weaknesses. Within the context of an investigative interview, if police repeat many demands in a rapid manner the individual with ASD may have significant difficulty in being able to process anything that they have been told to do given that the instructions were said far too fast for them to be able to fully process (Kroncke et al., 2016). Additional time is often needed in order to allow time to process verbal information and to provide a response to a question (Crane & Maras, 2018; Murphy, 2018), which is typically referred to as ‘Asperger time’ (e.g. Jacobsen, 2003; Myles et al., 2005).

## Discussion

Until Mr Hall was interviewed by police, the eyewitness identification evidence indicated that Mr Easton’s killer was six feet tall, dark skinned, sturdy and likely right-handed. Mr Hall’s police interviews, and the police interpretation of his responses and manner, led to a series of decisions and actions by police and the prosecutor, which culminated in Mr Hall being convicted of crimes he had not committed. In 2022, The Crown accepted that most of the content of Mr Hall’s police statements was unfairly obtained. The Crown emphasised the different standards, practicalities and methodologies that applied to police interviews in the 1980s. However, that does not satisfactorily explain what is apparent from the record: that Mr Hall’s police interviews were extremely lengthy; the interviews were conducted by multiple interviewing officers; Mr Hall was lied to by Police; the interviews occurred without Mr Hall having a lawyer or support person present; and questions were asked and comments and statements of opinion offered by interviewing officers. While the interviews would not be permissible by today’s standards, the Supreme Court unequivocally concluded that they fell short of what was tolerated in the 1980s, too. The Crown now accepts that a substantial miscarriage of justice occurred in Mr Hall’s case. It is imperative that there is an understanding of the influence that having a diagnosis of ASD can have on the investigative interview process (e.g. Murphy, 2018; Nesca & Dalby, 2013). By not recognising the presence of ASD in Mr Hall at this stage of his involvement in the criminal justice system, there are a number of aspects of his interview – the questions posed to him and his responses to these – that could be interpreted as being evidence of evasiveness, remorse, lack of empathy and guilt by both the investigating interviewers and the jury and judge when the evidence from this interview was later presented in court. Cross examination of some of the police officers who carried out their investigative interview with Mr Hall in 1985 revealed that he had exhibited some evidence of intellectual impairment – specifically, in his ability to spell. There are a number of clear indications during the police interviews (raised in the court proceedings) that Mr Hall was potentially impaired intellectually and therefore a potentially vulnerable individual. The Crown accepts and considers it relevant that Mr Hall has an intellectual vulnerability (note: Mr Hall does not fulfil the definition for intellectual disability). This was apparent during Mr Hall’s trial, and was also referenced in his first appeal, with varying descriptions of him as ‘backward intellectually’ and ‘an intellectually deprived person’.

It is well established in the literature that autistic adolescents and adults have frequent contact with police. For instance, one survey of 35 Canadian adults with ASD aged between 18 and 65 years found that 80% reported at least one interaction with police in their lifetime. Findings also revealed that 39% reported four to nine interactions with police, and 14% reported 10 or more interactions (Salerno & Schuller, 2019 – see also, Salerno-Ferraro & Schuller, 2020). In another study, around 20% of youth with ASD were found to have interacted with law enforcement officers by the time they were 21 years old (Rava et al., 2017). In a prospective Canadian study, which followed a sample of 284 adolescents and adults with ASD over 12 to 18 months, found that around 16% of individuals were reported to have some form of police involvement during the study period (Tint et al., 2017). Research has also shown that members of the ASD community (i.e. parents and adults with ASD) have expressed dissatisfaction with respect to their experiences with police (Crane et al., 2016; Helverschou et al., 2018).

The discussion demonstrates the potential for individuals with ASD to be drawn into the investigative process, for them to be vulnerable in this context and for negative outcomes to occur. This underscores the need for police officers to be aware of such issues and adapt their investigative interviewing techniques accordingly. A growing number of studies have highlighted deficits in this respect and the need for better formalised training on ASD for police officers and other law enforcement professionals (Gardner et al., 2019). There is evidence that members of the ASD community (e.g. parents and adults with ASD) do not experience satisfying interactions with police (Crane et al., 2016; Helverschou et al., 2018). Crane and colleagues explored the experiences and views of ASD from 394 police officers from England and Wales in the UK, using an online survey. Participants were asked questions regarding the measures and adjustments they had used when interviewing someone with a diagnosis of ASD (a total of 199 police officers responded to this question). The findings revealed that the adjustments that police made most frequently included: avoiding long-winded or multiple-part questions (92%); allowing extra time to process questions (91%); and being mindful of the vocabulary used (89%). Such adaptations indicate that the officers have some appreciation of ASD and common challenges with communication and cognitive processing. Participants were also asked in the study how easy or difficult it was to make these adjustments (a total of 175 participants responded to this question). Nearly half (49%) of police officers reported it was easy to make these adjustments, with 19% said they found it difficult (whilst 32% were neutral). In 47 open-ended responses, officers expanded on why this was the case, specifically identifying time constraints and a lack of training as factors that were considered to be significant barriers to enabling them to make appropriate adaptations and adjustments to support individuals with ASD in investigative interviews. Crane and colleagues found that only 42% of officers were satisfied with how they had worked with individuals with ASD. Even though 37% of officers had received general training on ASD, they identified a need for training that was tailored to specific policing roles (for example, frontline officers and detectives). Crane and colleagues also explored the experiences of the ASD community (31 adults with ASD, 49 parents) who were found to be largely dissatisfied with their experience of the police and also recommended the need for improved police training on ASD. A more recent pilot survey of 51 police officers in the United States conducted by Christiansen et al. (2023) found deficits in police training. They found that 52.9% reported previous ASD training, 34.8% reported personal experience with ASD, and 56.9% endorsed low overall knowledge of ASD. The need for formalised training in ASD for police officers and other law enforcement professionals has also been emphasised by an increasing number of studies (e.g. Gardner et al., 2019; Gibbs & Haas, 2020; Haas & Gibbs, 2021; Holloway et al., 2020; Love et al., 2021 – see also, Young & Brewer, 2020).

As in the case with Mr Alan Hall, adults with autism may be erroneously judged as deceptive or lacking credibility as a result of the unexpected and atypical behaviours they display. A recent study carried out by Lim et al. (2022) investigated this. In their study, 30 individuals with autism and 29 neurotypical individuals participated in video-recorded interviews, and they measured their demonstration of a number of features commonly associated with autism, including: gaze aversion, repetitive body movements, literal interpretation of figurative language, poor reciprocity and flat affect. The 1410 participants in their study viewed one of these videos and rated their perception of the individual’s truthfulness or credibility. Findings showed that individuals with autism were perceived by participants as more deceptive and less credible than the neurotypical individuals when telling the truth. However, the researchers emphasise that this relationship was influenced by the overall presentation of the individual rather than the presence of any of the target behaviours such as: levels of gaze aversion, repetitive body movements, literal interpretation of figurative language, poor reciprocity or flat affect (Lim et al., 2022).

The above discussion highlights that even though we have come a long way in police investigating interviewing since Mr Hall was interviewed by police in 1985, there still is a need for more police training around autism. Our knowledge and understanding of autism in forensic contexts are constantly increasing though empirical research, and so on, and this needs to be integrated into police training around autism. For instance, Dr David Murphy, a Chartered Forensic and Consultant Clinical Neuropsychologist in the United Kingdom, provided an insightful discussion of how to interview individuals with ASD in forensic settings (Murphy, 2018). Murphy developed a checklist of areas that need to be taken into consideration when interviewing individuals with a diagnosis of ASD, which we recommend law enforcement professionals integrate into their training. Some of the areas that Murphy has in his checklist include: personal safety; sensory issues; difficulties with reciprocal social communication; cognitive style and co-morbidity. Additionally, Salerno-Ferraro and Schuller (2020) provided a summary of recommendations that may be easily implemented by police officers and other law enforcement professionals during their interactions with individuals with ASD:

- Individuals with ASD should be allowed to engage in typical autistic behaviours such as self-stimulation as these can be self-soothing and may help in facilitating interactions and de-escalation. Some examples of typical autistic behaviours include repetitive movements and fidgeting which is sometimes referred to as ‘stimming’ (self-stimulation which helps soothe the individual). It is also recommended that you do not force the individual with autism to make or maintain eye contact.
- It is critical that the interviewer uses unambiguous, clear language when communicating with individuals with ASD. They also need to be patient and also ensure that they slow down and allow the individual more time to respond to the question. If the individual is unable to answer the question, the professional is recommended to rephrase the question, or offer an alternative communication tool (e.g. a notebook). They could also offer to call an intermediary (e.g. a parent, family member or caregiver) on behalf of the individual with ASD.
- It is helpful to maintain a calm demeanour and it can also be helpful to carry out the interaction/interview in a minimal sensory environment. A minimal sensory environment can be achieved by switching off any sirens (both lights and sounds), avoiding the use of bright lights (e.g. flashlights, headlights), and speaking in a soft voice. When possible, the professional(s) is also recommended to adopt a ‘hands-off’ approach due to the fact that touching an individual with ASD may further escalate the situation.
- If it is suspected that the individual has ASD, ask them. Tell them that they have the right to disclose any disabilities or mental health conditions, and that by doing so, it can help their situation (Salerno-Ferraro & Schuller, 2020).

## Conclusion

The impact of Mr Hall’s ASD and his resulting vulnerabilities cascaded through the police investigation and prosecution. The police approach to the interviews and their interpretation of Mr Hall’s responses and manner not only altered the course of the police investigation but were also catalysts for the prosecution manipulating and withholding evidence to secure Mr Hall’s convictions.

It is imperative that ASD is identified and taken into consideration as early as possible during the criminal justice process (Cooper & Allely, 2017). Failure to do so may contribute to a miscarriage of justice occurring, as it did for Alan Hall.

## Ethical standards

### Declaration of conflicts of interest

C. S. Allely was an expert witness in Alan Hall’s miscarriage of justice appeal.

T. McKinnel was lead investigator in Alan Hall’s miscarriage of justice appeal.

N. Chisnall was Alan Hall’s counsel in the Supreme Court.

## Ethical approval

This article does not contain any studies with human participants or animals performed by any of the authors.
